# Blood pressure in Warmblood horses before and during a euglycemic-hyperinsulinemic clamp

**DOI:** 10.1186/s13028-016-0247-y

**Published:** 2016-10-20

**Authors:** Katarina E. A. Nostell, Sanna S. Lindåse, Johan T. Bröjer

**Affiliations:** Department of Clinical Sciences, Swedish University of Agricultural Sciences, Box 7054, 750 07 Uppsala, Sweden

**Keywords:** Insulin resistance, Horse, Blood pressure, Euglycemic hyperinsulinemic clamp

## Abstract

**Background:**

Insulin resistance (IR) in humans is related to hypertension and impaired vasodilation. Insulin administration has been shown to lower blood pressure both in insulin resistant as well as in insulin sensitive individuals. The aim of the study was to investigate the association between insulin sensitivity and alterations in blood pressure in healthy horses before and after a euglycemic-hyperinsulinemic clamp (EHC). A 3-h EHC was performed in 13 healthy horses (11 mares, 2 geldings). Blood samples for measurement of plasma glucose and insulin were collected before the start of the EHC, every 10 min during the EHC and immediately after the EHC. Mean, systolic- and diastolic blood pressure was measured before and during the final 10 min of the EHC using an indirect high-definition oscillometric monitor (HDO, horse model) applied to the middle of the coccygeal artery. Five consecutive measurements were made in each horse and on each occasion. Insulin and glucose data from the EHC were used to calculate the mean rate of glucose disposal per unit of insulin during steady state (M/I ratio). Insulin resistance was defined as a M/I ratio <5 mg/kg/min/mUL (Lindåse et al. in Am J Vet Res 77:300–309, 2016).

**Results:**

Insulin administration decreased systolic, diastolic and mean arterial pressure in all horses. The M/I ratio for all horses was negatively correlated with the decrease in systolic blood pressure (r^2^ = 0.55, P = 0.004) and mean arterial pressure (r^2^ = 0.31, P = 0.048) but not diastolic blood pressure (r^2^ = 0.12, P = 0.26). Eight horses were defined as insulin resistant (IR) and five horses had normal insulin sensitivity. The five horses with normal insulin sensitivity showed a greater decrease in systolic blood pressure (−17.0 ± 7.4 vs. −3.4 ± 4.6 mmHg, P = 0.001) and MAP (19.2 ± 14.7 vs. 6.9 ± 8.7 mmHg, P = 0.04) than IR horses. There was no difference in the decrease in diastolic blood pressure between groups (16 ± 12.8 vs. 8.9 ± 12.1 mmHg, P = 0.17).

**Conclusions:**

This study indicates that there is a relationship between insulin sensitivity and systolic and MAP in horses. However, studies on a larger number of horses are needed to confirm this association.

## Background

Insulin is an important anabolic hormone, especially when it comes to glucose metabolism, but it also has important cardiovascular regulatory properties that help to regulate blood pressure during normal physiological conditions [1–3]. Insulin can act both as a vasodilator and a vasoconstrictor by acting at different levels of the cardiovascular system [1, 2]. For instance, insulin stimulates the sympathetic nervous system by potentiating the response to noradrenaline leading to vasoconstriction but it also potentiates vasodilation by enhancing the response to acetylcholine (AC) [1]. In addition to this it also acts on the endothelial cells and stimulates the release of nitric oxide (NO) as well as endothelin (ET), where NO acts as a vasodilator and ET is a potent vasoconstrictor [2]. Moreover insulin activates the renin-angiotensin-aldosterone system (RAAS) and thereby increases the retention of sodium and water in the distal tubuli of the nephron [1, 3]. Altogether this helps to balance blood pressure during normal physiological conditions.

In humans it is well known that conditions causing insulin resistance (IR) and hyperinsulinemia (HI) are associated with hypertension [4–6]. The exact mechanisms behind this are not known. However, studies in humans and animals have indicated that IR and HI increase the sympathetic tone by stimulating the release of NA [7]. There are indications that during IR and HI the response to AC and the release of NO are impaired whereas the stimulatory effect on both ET and NA remains [8, 9]. There are also increasing evidence that obesity, which is often associated with IR, further stimulates the RAAS and causes an increased retention of sodium and water which in turn increases the circulating blood volume [1, 3]. All of this promotes a rise in blood pressure and in the long run causes hypertension.

From human studies it is known that insulin administration lowers systemic blood pressure both in IR as well as insulin sensitive individuals and that this likely is related to a vasodilatory effect [1, 10, 11]. In many of these studies blood pressure was measured when insulin was administered during a euglycemic hyperinsulinemic clamp (EHC). Whether insulin administration influences blood pressure in horses is not known. The major aim of the present study was therefore to investigate the association between insulin administration and alterations in blood pressure before and after a EHC in horses. There are also indications that insulin resistant individuals have a blunted insulin induced vasodilation [12]. An additional aim was therefore to see if there are any indications that IR horses also have an altered response to the action of insulin.

## Methods

All procedures were sanctioned by the Ethical Committee for Animal Experiments, Uppsala, Sweden (C304/12).

### Horses

Thirteen horses were included in the study, 11 Standardbreds (9 mares and 2 geldings) and 2 Warmblood mares. The average body weight and age of the horses were 505 ± 49 kg (429–572) and 13 ± 4 years (6–20) respectively. The mean body condition score (BCS) was 5.5 ± 0.8 (4.5–7) on a scale from 1 to 9, where 1 is considered to be extremely emaciated and 9 extremely fat [13]. Mean Cresty Neck Score (CNS) was 2.5 ± 0.7 (1.5–4) on a scale from 0 to 5, where 0 is no visual appearance of crest and 5 a crest that is so large it permanently falls over to one side [14]. The Standardbreds were all owned by the Department of Clinical Sciences whereas the Warmblood horses were privately owned. The Standardbreds were sedentary horses whereas the Warmbloods were riding horses that were exercised at a regular basis. The horses had no history of laminitis and none of the horses had any signs of disease upon the clinical examination that was performed before the start of the study. All horses were kept in box stalls with a daily turnout in a paddock. The horses were acclimatised to the environment and were accustomed to the HDO monitor (high definition oscillometry monitor, horse model) by applying the device to the tail once daily on two consecutive days prior to the EHC. The Warmblood horses underwent an acclimatisation period of 3 days before the experiment. All horses had ACTH concentrations within the normal reference range for the season (<7 pmol/L).

### Experimental design

#### EHC

The day before the EHC, the horses were weighed, and catheters (2.0 × 105 mm Vygon, Ecouen, France) were introduced into both jugular veins after clipping and antiseptic preparation. One of the catheters was used for infusion of glucose and insulin and the other for the collection of blood samples. The horses were kept off feed for 12 h before the start of the EHC but were allowed free access to water.

Blood samples for analysis of plasma glucose and insulin were collected before the start of the EHC (−10, −5, and −1 min). The horses then received a continuous infusion of insulin (Humulin Regular, Eli Lilly Sweden AB, Solna, Sweden) at a rate of 3 mU/kg/min together with a 50 % solution of glucose, were the infusion rate was adjusted in order to keep blood glucose at a euglycemic level (defined as 5 mmol/L). Blood glucose was analysed every 5 min throughout the EHC using a hand held glucometer (Accu-Check Aviva, Roche Diagnostics Scandinavia AB, Bromma, Sweden) previously validated (in house) for use in horses. The glucose infusion rate was adjusted if the blood glucose concentration deviated by more than 0.2 mmol/L from euglycemia. Blood samples were collected before the start of the EHC (−10 and −1 min), every 10-min throughout the EHC for subsequent determination of plasma glucose (10 min intervals) and plasma insulin (20 min intervals). The blood samples were kept on ice until centrifuged at 2770×*g* for 10 min (2770×*g*), within 15 min after sampling, and stored at −80 °C until analysis.

#### Blood pressure

Blood pressure recordings were obtained once before and during the last 10 min of the EHC with the horses standing in the box stall. Mean arterial- (MAP), systolic-, and diastolic blood pressure was measured with the horse in confinement of the box stall, using an indirect oscillometric device; HDO (Memo Diagnostic High Definition Oscillometry Monitor, horse model, S + B medVET GmbH, Babenhausen, Germany) applied to the middle of the coccygeal artery. This device has previously been evaluated for use in horses [15–17]. Five consecutive measurements were made. In each horse, the lowest and highest recorded value of the five consecutive blood pressure determinations were excluded and the mean, systolic and diastolic pressure was calculated from the three remaining determinations.

#### Analysis

Insulin resistance was defined as a M/I ratio <5 mg/kg/min × 10^3^/mUL [18]. Plasma glucose concentrations were measured with an enzymatic method using an automated clinical chemistry analyzer (YSI 2300 Stat Plus Analyzer, YSI Incorporated, Yellow Spring, Ohio). Plasma insulin concentrations were analysed with a commercially available ELISA for humans (Mercodia Insulin ELISA, Mercodia AB, Uppsala, Sweden) and a commercial insulin control kit [Mercodia Diabetes Antigen Control (Low, High)/Human, Mercodia AB, Uppsala, Sweden] horses [19]. All analyses of plasma glucose and insulin were performed in duplicate. The mean intra-assay CVs for glucose was 0.5 %, and for insulin 3.3 %, as determined from duplicate analyses.

#### Calculations and statistics

All data were analysed using the mixed model procedure in JMP^®^ Pro 11.0.0. (SAS Institute Inc., Cary, NC, USA). Results are expressed as mean ± SD. The change in blood pressure before and after the EHC was analysed using a paired t test. Regression analysis was performed and the correlation coefficient was calculated according to the method of Pearson. The residuals were normally distributed. A value of P < 0.05 was considered statistically significant.

The first 120 min of the EHC were considered an equilibration period. Data from the final 60 min (steady-state) was used for calculations of M and M/I ratio.

The M value was calculated for each 10-min interval by the following equation:

$${\text{M}} = {\text{GIR}} - {\text{SC}},$$

where GIR is the glucose infusion rate and SC is the space correction. The M is the mean of the six M values for each 10-min interval during steady state. The space correction represented an adjustment for glucose that has been added to or removed from the glucose space other than by metabolism.

The SC was calculated from the equation:

$${\text{SC}} = {{\left( {{\text{G}}2 - {\text{G}}1} \right) \times 0.19} \mathord{\left/ {\vphantom {{\left( {{\text{G}}2 - {\text{G}}1} \right) \times 0.19} {\text{T}}}} \right. \kern-0pt} {\text{T}}},$$

where G1 and G2 are plasma glucose concentrations at the beginning and end of the time interval, T is the time interval (10 min).

The M/I ratio was calculated for each 20-min interval where I is the plasma insulin concentration for the interval. The M/I ratio is the mean of the three M/I ratios for each 20-min interval during steady state.

## Results

Preprandial insulin concentrations were 9.3 ± 5.2 mU/L and glucose 5.2 ± 0.7 mmol/L. The horses had M values of on average 3.1 ± 0.8 mg/kg/min and M/I ratios of 5.3 ± 0.6 (mg/kg/min × 10^3^)/(mU/L).

### Blood pressure and insulin administration

**Fig. 1:**
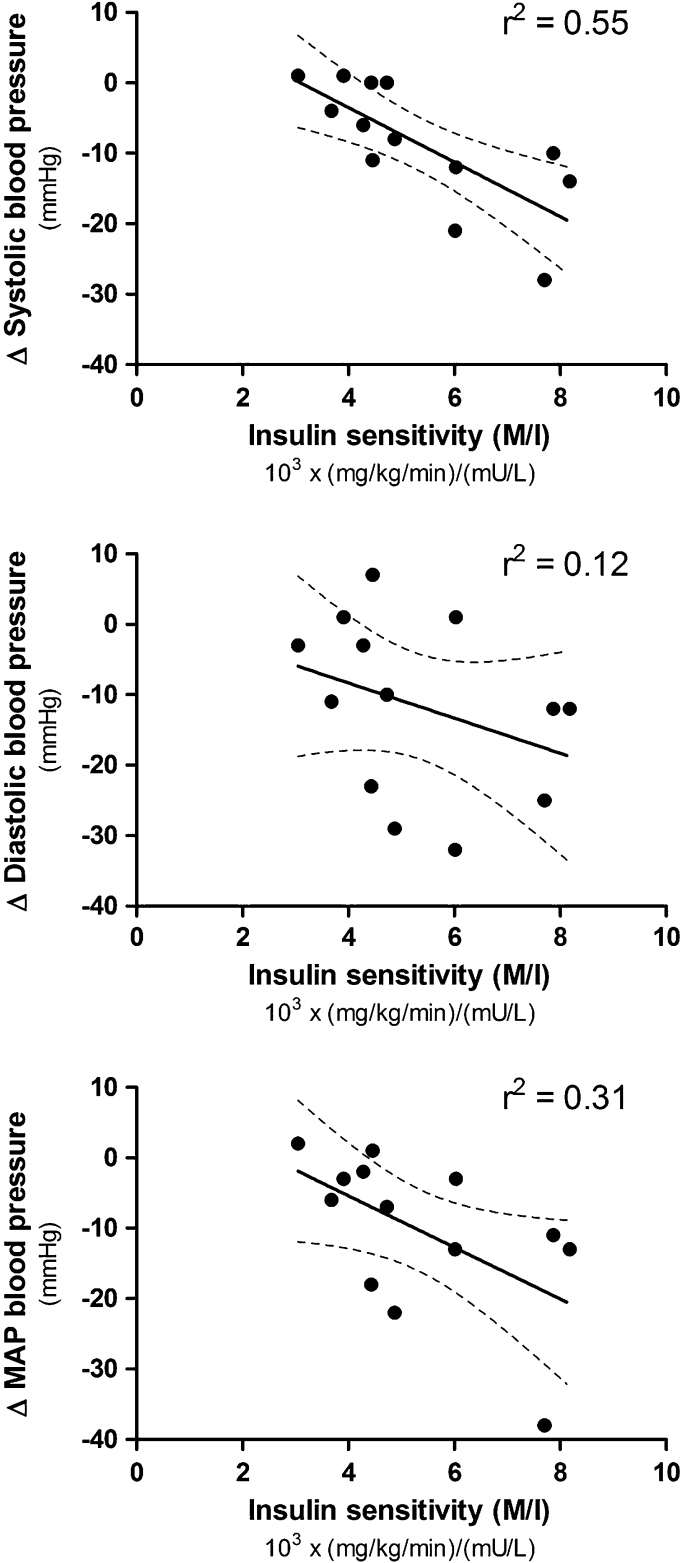
Relationship between the change in (delta) values for systolic- (*upper panel*), diastolic- (*middle panel*) and MAP- (*lower panel*) pressure and insulin sensitivity (M/I ratio) in 13 healthy horses before and after a euglycemic hyperinsulinemic clamp, P ≤ 0.05

Resting mean values was 113 ± 11 mmHg for systolic blood pressure, 69 ± 13 mmHg for diastolic blood pressure and 87 ± 16 mmHg for MAP for all horses. Insulin administration decreased systolic- (104 ± 7 mmHg, P = 0.004), diastolic- (57 ± 10 mmHg, P = 0.006) and MAP (75 ± 8 mmHg, P = 0.006) in all horses. The M/I ratio for all horses was negatively correlated with the decrease in systolic blood pressure (r^2^ = 0.55, P = 0.004) and MAP (r^2^ = 0.31, P = 0.048) but not diastolic blood pressure (r^2^ = 0.12, P = 0.26), see Fig. 1. This means that 55 % of the decrease in systolic blood pressure could be related to the effects of insulin in this situation. An increase in M/I ratio with one unit caused a decrease in blood pressure of 3.74 mmHg. The M value for all horses also showed a negative correlation with the decrease in systolic- (r^2^ = 0.37, P = 0.027) and MAP (0.31, P = 0.049) but not diastolic blood pressure (r^2^ = 0.08, P = 0.36),

Eight horses were defined to be insulin resistant [M/I ratio 4.2 ± 0.6 (mg/kg/min × 10^3^)/(mU/L)] and five horses were defined as insulin sensitive [M/I index 7.2 ± 1.0 (mg/kg/min × 10^3^)/(mU/L)]. The five horses with normal insulin sensitivity showed a greater decrease in systolic blood pressure (−17.0 ± 7.4 vs. −3.4 ± 4.6 mmHg, P = 0.001) and MAP (19.2 ± 14.7 vs. 6.9 ± 8.7 mmHg, P = 0.04) than IR horses. There was no difference in the decrease in diastolic blood pressure between groups (16 ± 12.8 vs. 8.9 ± 12.1 mmHg, P = 0.17).

## Discussion

This study shows that systolic, diastolic as well as MAP decreased in horses during insulin administration when blood glucose was kept at a euglycemic level. This is in agreement with previous studies in humans where decreased blood pressures were seen in association with insulin administration [11, 20]. The reason for the decrease in blood pressure in the present study is not known but previous studies in humans and dogs have shown that insulin administration promotes peripheral vasodilation [1, 10, 11, 20, 21]. As the horses in the present study showed no signs of anxiety and were accustomed to blood pressure recordings it is reasonable to assume that the decrease seen in blood pressure at the end of the EHC is the result of insulin induced vasodilation. However, in some of the previously mentioned studies no change in blood pressure was seen after insulin administration despite marked vasodilation [10, 13]. The reason for this discrepancy in results could be the highly variable vasodilatory effect of insulin seen between different individuals [10, 22]. Blood pressure was measured using a non-invasive technique, a method that that is likely to give more heterogeneous results than an invasive measurement and this could of course also influence the obtained results. The degree of vasodilation is also dependent on the dosage and duration of insulin administration as well as the insulin sensitivity of the studied individual. The time course of insulin’s action from administration to the onset of vasodilation also varies depending on the dosage used. Most studies have used an insulin infusion rate of 1 mU/kg/min in order to achieve circulating insulin concentrations of 70–100 μU/ml and with this infusion rate it takes 40–60 min to get half-maximal rates of blood flow [23]. In the present study, a higher infusion rate (3 mU/kg/min) was used which is likely to have caused an earlier onset of vasodilatation. It is therefore reasonable to assume that changes in blood pressure could have occurred earlier during the EHC in the horses in the present study but as blood pressure measurements were only made during the last 10 min of the EHC and not repeatedly during the EHC this cannot be verified.

This study also indicates that horses with insulin resistance, in similarity to humans, have a altered response to the cardiovascular effects of insulin [11]. However, the study is done on a small number of horses and the fact that blood pressure was only measured once during the EHC warrants studies in a larger number of horses and using more frequent blood pressure recordings. In humans, the association between IR is stronger for diastolic than for systolic blood pressure whereas in horses, the correlation was strongest for IR and systolic blood pressure and no correlation was found between IR and the decrease in diastolic pressure [11]. The reason for this discrepancy in results is likely related to that the diastolic pressure is a reflection of a change in laminar flow that is more difficult to detect than systolic blood pressure, especially in animals that have hair coating and a thicker skin than humans. This is supported by the results from a previous study using the HDO device were the CV was higher for the diastolic- than the systolic blood pressure measurements [15].

To our knowledge, this is the first in vivo study that indicates that horses with IR have a decreased vasodilatory response to insulin but it does not explain the mechanisms that cause this altered cardiovascular response as this is beyond the scope of this study. There are however in vitro studies performed on vessels from euthanized horses that have looked at the cardiovascular effects of insulin on vessel function [24–27]. These studies have shown that insulin causes vasorelaxation and that short-term hyperinsulinemia induces an altered vascular relaxation response [24–26]. In vivo studies in humans and animals have indicated that IR and HI increase the sympathetic tone by stimulating the release of NA [7]. There are indications that during IR and HI states the response to AC and the release of NO are impaired whereas the stimulatory effect on both ET and NA remains [8, 24]. There are also increasing evidence that obesity, which is often associated with IR, further stimulates the RAAS and causes an increased retention of sodium and water which in turn increases the circulating blood volume.

All of these factors promote a rise in blood pressure and in the long run cause hypertension. An altered vascular response to insulin leading to an increase in vascular tone could perhaps explain the described increase in systemic blood pressure seen in previously laminitic ponies [28]. None of the horses were hypertensive although they had varying degree of insulin sensitivity. This is likely related to the fact all of the horses were healthy and of breeds with no known predisposition for IR but with varying degree of IR due to inactivity. In humans it is discussed that both the degree of insulin sensitivity and the level of hyperinsulinemia may affect blood pressure through separate pathways. Consequently, the actual blood pressure levels will depend on which pressor or depressor action of insulin is resistant or sensitive. There are studies that have implied that IR and not HI is associated with hypertension in healthy individuals and individuals with diabetes, and that blood pressure is lower in IR individuals with HI than IR normoinsulinemic individuals [5, 6, 29]. None of the horses in the present study were HI but all had varying degree of IR, which could imply that the mechanism is different in horses compared to man. However, studies on a larger group of horses are needed to confirm this.

## Conclusions

This study indicates that there is a relationship between insulin sensitivity and systolic and MAP in horses. However, studies on a larger number of horses are needed to confirm this association.

## Abbreviations

AC: acetylcholine
BCS: body condition score
CNS: Cresty Neck Score
CV: coefficient of variation
EHC: euglycemic-hyperinsulinemic clamp
ET: endothelin
HDO: high definition oscillometry
HI: hyperinsulinemia
IR: insulin resistance
M value: calculated measurement for metabolised glucose
MAP: mean arterial blood pressure
M/I ratio: calculated measurement for metabolised glucose/unit plasma insulin
NA: noradrenaline
NO: nitric oxide
RAAS: renin-angiotensin system

## References

[1] Anderson EA, Hoffman RP, Balon TW, Sinkey CA, Mark AL (1991). Hyperinsulinemia produces both sympathetic neural activation and vasodilation in normal humans. J Clin Invest.

[2] Polderman K, Stehouwer C, van Kamp G, Gooren L (1996). Effects of insulin infusion on endothelium-derived vasoactive substances. Diabetologia.

[3] Natali A, Quiñones Galvan A, Santoro D, Pecori N, Taddei S, Salvetti A (1993). Relationship between insulin release, antinatriuresis and hypokalemia after glucose ingestion in normal and hypertensive man. Clin Sci.

[4] Modan M, Halkin H, Almog S, Lusky A, Eshkol A, Shefi M (1985). Hyperinsulinemia: a link between hypertension, obesity and glucose intolerance. J Clin Invest.

[5] Ferrannini E, Natali A, Capaldo B, Lehtovirta M, Jacob S, Yki-Järvinen H, Capaldo B, Lehtovirta M, Jacob S, Yki-Järvinen H (1997). Insulin resistance, hyperinsulinemia, and blood pressure: role of age and obesity. European Group for the Study of Insulin Resistance (EGIR). Hypertension.

[6] Pollare T, Lithell H, Berne C (1990). Insulin resistance is a characteristic of primary hypertension independent of obesity. Metabolism.

[7] Rowe J, Young J, Minaker K, Stevens A, Pallotta J, Landsberg L (1981). Effect of insulin and glucose infusions on sympathetic nervous system activity in normal man. Diabetes.

[8] Forte P, Copland M, Smith LM, Milne E, Sutherland J, Benjamin N (1997). Basal nitric oxide synthesis in essential hypertension. Lancet.

[9] Ferri C, Laurenti O, Bellini C, Faldetta MR, Properzi G, Santucci A (1995). Circulating endothelin-1 levels in lean non-insulin-dependent diabetic patients. Influence of ACE inhibition. Am J Hypertens.

[10] Laakso M, Edelman S, Brechtel G, Baron A (1990). Decreased effect of insulin to stimulate skeletal muscle blood flow in obese man. A novel mechanism for insulin resistance. J Clin Invest.

[11] Heise T, Magnusson K, Heinemann L, Sawicki P (1998). Insulin resistance and the effect of insulin on blood pressure in essential hypertension. Hypertension.

[12] Feldman RD, Bierbrier GS (1993). Insulin-mediated vasodilation: impairment with increased blood pressure and body mass. Lancet.

[13] Henneke DR, Potter GD, Kreider JL, Yeates BF (1983). Relationship between condition score, physical measurements and body fat percentage in mares. Equine Vet J.

[14] Carter RA, Geor RJ, Burton Staniar W, Cubitt TA, Harris PA (2009). Apparent adiposity assessed by standardised scoring systems and morphometric measurements in horses and ponies. Vet J.

[15] Söder J, Bröjer J, Nostell K (2012). Interday variation and effect of transportation on indirect blood pressure measurements, plasma endothelin-1 and serum cortisol in Standardbred and Icelandic horses. Acta Vet Scand.

[16] Walders W, Gehlen H (2014). Noninvasive blood pressure measurement using high definition oscillometry in horses with heart diseases. Tierarztl Prax Ausg G Grosstiere Nutztiere.

[17] Tünsmeyer J, Hopster K, Feige K, Kästner SB (2015). Agreement of high definition oscillometry with direct arterial blood pressure measurement at different blood pressure ranges in horses under general anaesthesia. Vet Anaesth Analg.

[18] Lindåse S, Nostell K, Müller C, Jensen-Waern M, Bröjer J (2016). Effects of diet-induced weight gain and turnout to pasture on insulin sensitivity in moderately insulin-resistant horses. Am J Vet Res.

[19] Öberg J, Bröjer J, Wattle O, Lilliehöök I (2011). Evaluation of an equine-optimized enzyme-linked immunosorbent assay for serum insulin measurement and stability study of equine serum insulin. Comp Clin Pathol..

[20] Baron A, Bechtel G (1993). Insulin differentially regulates systemic and skeletal muscle vascular resistance. Am J Physiol.

[21] Brands MW, Mizelle HL, Gaillard CA, Hildebrandt DA, Hall JE (1991). The hemodynamic response to chronic hyperinsulinemia in conscious dogs. Am J Hypertens.

[22] Laakso M, Edelman S, Brechtel G, Baron A (1992). Impaired insulin mediated skeletal muscle blood flow in patients with NIDDM. Diabetes.

[23] Prager R, Wallace P, Olefsky J (1986). In vivo kinetics of insulin action on peripheral glucose disposal and hepatic glucose output in normal and obese subjects. J Clin Invest.

[24] Gauff F, Patan-Zugaj B, Licka TF (2013). Hyperinsulinaemia increases vascular resistance and endothelin-1 expression in the equine digit. Equine Vet J.

[25] Venugopal CS, Eades S, Holmes EP, Beadle R (2011). Insulin resistance in equine digital vessel rings: an in vitro model to study vascular dysfunction in equine laminitis. Equine Vet J.

[26] Keen JA, McGorum BC, Hillier C, Nally JE (2013). Short-term incubation of equine laminar veins with cortisol and insulin alters contractility in vitro: possible implications for the pathogenesis of equine laminitis. J Vet Pharmacol Ther.

[27] Wooldridge AA, Waguespack RW, Schwartz DD, Venugopal CS, Eades SC, Beadle RE (2014). Vasorelaxation responses to insulin in laminar vessel rings from healthy, lean horses. Vet J.

[28] Bailey SR, Habershon-Butcher JL, Ransom KJ, Elliot J, Menzies-Gow NJ (2008). Hypertension and insulin resistance in a mixed-breed population of ponies predisposed to laminitis. Am J Vet Res.

[29] Bonora E, Capaldo B, Perin PC, Del Prato S, De Mattia G (2008). Hyperinsulinemia and insulin resistance are independently associated with plasma lipids, uric acid and blood pressure in non-diabetic subjects. The GISIR database. Nutr Metab Cardiovasc Dis.

